# Automated GMP compliant production of [^18^F]AlF-NOTA-octreotide

**DOI:** 10.1186/s41181-019-0084-1

**Published:** 2020-01-29

**Authors:** Térence Tshibangu, Christopher Cawthorne, Kim Serdons, Elin Pauwels, Willy Gsell, Guy Bormans, Christophe M. Deroose, Frederik Cleeren

**Affiliations:** 1 Radiopharmaceutical Research, Department of Pharmaceutical and Pharmacological Sciences, KU Leuven, Herestraat 49 Box 821, 3000 Leuven, Belgium; 2 Nuclear Medicine, University Hospitals Leuven, Leuven, Belgium; 3 Nuclear Medicine and Molecular Imaging, Department of Imaging and Pathology, KU Leuven, Leuven, Belgium; 4 Biomedical MRI/MoSAIC, Department of Imaging and Pathology, Biomedical Sciences Group, KU Leuven, Leuven, Belgium

**Keywords:** AlF-NOTA-octreotide, Fluorine-18, PET, Al^18^F, Octreotide, Somatostatin

## Abstract

**Background:**

Gallium-68 labeled synthetic somatostatin analogs for PET/CT imaging are the current gold standard for somatostatin receptor imaging in neuroendocrine tumor patients. Despite good imaging properties, their use in clinical practice is hampered by the low production levels of ^68^Ga eluted from a ^68^Ge/^68^Ga generator. In contrast, ^18^F-tracers can be produced in large quantities allowing centralized production and distribution to distant PET centers. [^18^F]AlF-NOTA-octreotide is a promising tracer that combines a straightforward Al^18^F-based production procedure with excellent in vivo pharmacokinetics and specific tumor uptake, demonstrated in SSTR2 positive tumor mice. However, advancing towards clinical studies with [^18^F]AlF-NOTA-octreotide requires the development of an efficient automated GMP production process and additional preclinical studies are necessary to further evaluate the in vivo properties of [^18^F]AlF-NOTA-octreotide. In this study, we present the automated GMP production of [^18^F]AlF-NOTA-octreotide on the Trasis AllinOne® radio-synthesizer platform and quality control of the drug product in accordance with GMP. Further, radiometabolite studies were performed and the pharmacokinetics and biodistribution of [^18^F]AlF-NOTA-octreotide were assessed in healthy rats using μPET/MR.

**Results:**

The production process of [^18^F]AlF-NOTA-octreotide has been validated by three validation production runs and the tracer was obtained with a final batch activity of 10.8 ± 1.3 GBq at end of synthesis with a radiochemical yield of 26.1 ± 3.6% (dc), high radiochemical purity and stability (96.3 ± 0.2% up to 6 h post synthesis) and an apparent molar activity of 160.5 ± 75.3 GBq/μmol. The total synthesis time was 40 ± 3 min. Further, the quality control was successfully implemented using validated analytical procedures. Finally, [^18^F]AlF-NOTA-octreotide showed high in vivo stability and favorable pharmacokinetics with high and specific accumulation in SSTR2-expressing organs in rats.

**Conclusion:**

This robust and automated production process provides high batch activity of [^18^F]AlF-NOTA-octreotide allowing centralized production and shipment of the compound to remote PET centers. Further, the production process and quality control developed for [^18^F]AlF-NOTA-octreotide is easily implementable in a clinical setting and the tracer is a potential clinical alternative for somatostatin directed ^68^Ga labeled peptides obviating the need for a ^68^Ge/^68^Ga-generator. Finally, the favorable in vivo properties of [^18^F]AlF-NOTA-octreotide in rats, with high and specific accumulation in SSTR2 expressing organs, supports clinical translation.

## Background

Neuroendocrine tumors (NET) are neuronal and endocrine tissue neoplasms characterized by the inappropriate release of growth factors, cytokines, hormones or neurotransmitters (Van Binnebeek et al. 2011). NETs most often correspond to gastro-intestinal cancers, however, organs such as the lungs, gonads or adrenal glands may be affected as well. Molecular imaging plays a key role in the clinical management of NETs, both for diagnosis, recurrence detection, therapy follow-up and therapy selection (Barrio, M., et al. 2017). Due to nonspecific clinical signs, NETs are often only detected at an advanced disease stage with distant metastasis.

Somatostatin receptors (SSTRs) are validated biomarkers in the management of NETs. The short biological half-life of the endogenous ligand somatostatin (< 5 min) prevents pharmacological use of this peptide (Rai et al. 2015). Therefore, synthetic somatostatin analogs that are more resistant to peptidases, such as octreotide, were developed. Using radiolabeled somatostatin analogs, the presence of SSTRs on tumor cells may be exploited for molecular imaging and targeted radionuclide therapy (TRNT) for the treatment of unresectable neuroendocrine tumors (Pauwels et al. 2018; Deroose et al. 2016). Nowadays, ^68^Ga-labeled somatostatin analogs for positron emission tomography (PET), such as [^68^Ga]Ga-DOTATOC, [^68^Ga]Ga-DOTANOC and [^68^Ga]Ga-DOTATATE, are the gold standard offering improved sensitivity (Duijzentkunst et al. 2017).

However, implementation in clinical practice is often limited due to the low production batch yield related to the current generation of ^68^Ge/^68^Ga generators, the high cost of generators, and relatively short half-life (67.7 min) of gallium-68 that challenge centralized production and distribution (Deroose et al. 2016). An interesting alternative to generator-based production of ^68^Ga is the recent introduction of a liquid target for cyclotron-based production of ^68^Ga (Alves et al. 2017, Synowiecki et al. 2018). This strategy seems interesting and could reshuffle the cards.

Among β^+^- emitting radioisotopes, fluorine-18 is the most commonly used PET radionuclide and offers several physico-chemical and logistic advantages over gallium-68 (Kesch et al. 2017; Le Bars et al. 2006; Sanchez-Crespo et al. 2013; Cal-González et al. 2013). Large amounts of fluorine-18 can be produced with a medical cyclotron using the ^18^O(p,n)^18^F nuclear reaction and the half-life (109.8 min) is long enough to allow distribution to remote hospitals without an on-site cyclotron. However, direct radiofluorination of peptides is challenging. Therefore, the indirect radiolabeling strategy using prosthetic groups (e.g. [^18^F] fluoronicotinic acid tetrafluorophenyl-ester ([^18^F]Py-TFP)) (Olberg et al. 2010), is still the norm despite the time-consuming process and the significant challenges encountered for automation.

In contrast, the Al^18^F-labeling developed by McBride et al. combines the advantages of a chelator-based radiolabeling method with the unique properties of fluorine-18 (McBride et al. 2009). In this method, fluorine is firmly bound to Al^3+^ forming [^18^F] AlF which is then complexed by a suitable chelator, conjugated to a vector molecule of interest (e.g. octreotide) (Kumar and Ghosh 2018).

**Fig. 1:**
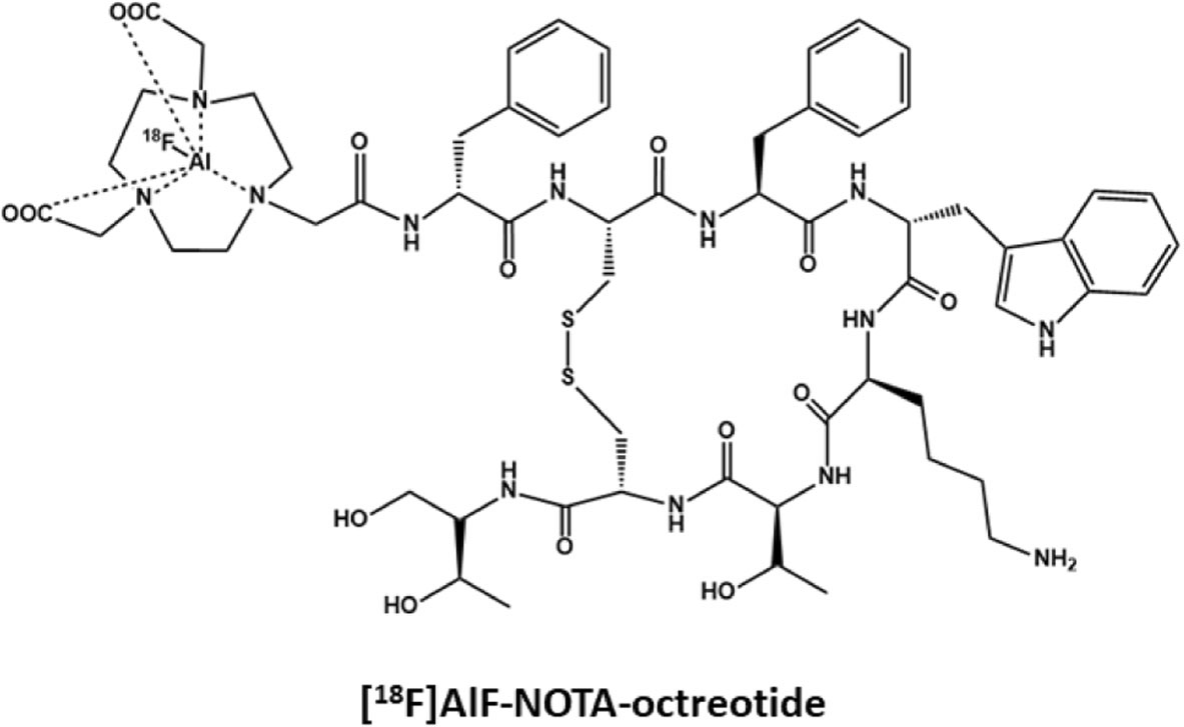
Chemical structure of [^18^F]AlF-NOTA-octreotide

[^18^F]AlF-NOTA-octreotide was developed by Laverman et al. as an alternative for ^68^Ga-labeled somatostatin analogs such as [^68^Ga]Ga-DOTATATE (Laverman et al. 2010; Laverman et al. 2012). The chemical structure of [^18^F]AlF-NOTA-octreotide is shown in Fig. 1. [^18^F]AlF-NOTA-octreotide has high affinity for SSTR (3.6 ± 0.6 nM) and demonstrated excellent in vitro stability (Laverman et al. 2010). Biodistribution studies in SSTR2 positive tumor mice showed high specific uptake of [^18^F]AlF-NOTA-octreotide in the tumor and in SSTR2-expressing tissues, such as the pancreas, adrenal glands and the stomach with little or no in vivo defluorination in mice (Laverman et al. 2012). However, in vivo metabolite studies are lacking. These promising preclinical results, the commercial availability of the precursor (GMP-grade) and the straightforward radiolabeling strategy, make [^18^F]AlF-NOTA-octreotide an ideal candidate for imaging NET in a clinical setting.

Allott et al. described recently a general Al^18^F-radiochemistry procedure on two automated radiosynthetizer platforms (Trasis AllinOne® and GE TRACERlab FX_FN_) and concluded that both systems have a great potential for the GMP productions of Al^18^F radiopharmaceuticals (Allott et al. 2017). They successfully produced [^18^F]AlF-NOTA-octreotide using this procedure but further optimization was required for routine clinical GMP production. Here we present the development of a robust, reproducible, simple and efficient GMP production process for [^18^F]AlF-NOTA-octreotide on the Trasis AllinOne® radiosynthesizer platform affording [^18^F]AlF-NOTA-octreotide in high batch activity and high apparent molar activity. Further, the validation of the analytical procedures and quality control of [^18^F]AlF-NOTA-octreotide are described, based on the monograph of “Gallium (^68^Ga) edotreotide injection” in the European Pharmacopoeia (Ph.Eur).

Although Laverman et al. obtained promising preclinical results with [^18^F]AlF-NOTA-octreotide in SSTR2 positive tumor mice, additional preclinical results with [^18^F]AlF-NOTA-octreotide are warranted to proceed with the clinical evaluation of [^18^F]AlF-NOTA-octreotide. Therefore, we performed plasma and urine radiometabolite studies in healthy rats and the pharmacokinetics and specificity of [^18^F]AlF-NOTA-octreotide were further assessed by performing baseline and blocking scans in healthy rats using μPET/MR.

## Methods

### General

All reagents and solvents were purchased from Sigma-Aldrich (Bornem, Belgium), Fluka (Bornem, Belgium), Fisher (Doornik, Belgium) or Acros Organics (Geel, Belgium) and were used without further purification. Octreotide acetate and the precursor NOTA-octreotide trifluoracetate were purchased from ABX advanced biochemical compounds (Radeberg, Germany). To the precursor solution containing 0.12 mg NOTA-octreotide in 0.1 M sodium acetate pH 4.1 (0.27 mL) and absolute ethanol, (EtOH) (0.3 mL), 30 μl of a freshly prepared sodium ascorbate solution (20 mg/mL sodium ascorbate (Ph. Eur, Fagron, Nazareth, Belgium) in HPCE grade water (Sigma Aldrich)) was added just before the radiolabeling. The formulation solution (EtOH/NaAsc 0.59% in NaCl 0.9% 1.1/11.9 V/V in water for injection), was prepared in advance under sterile conditions, bubbled with nitrogen, covered with aluminum foil and stored at 4 °C. All buffers used for radiolabeling were treated with chelex (chelex, 100 sodium form (Sigma Aldrich), 2 g/L, 30 min stirring at room temperature and filtration with a 0.45 μm polyamide filter (Sartorius Stedim Biotech, Göttingen, Germany)).

Fluorine-18 was produced on site using a cyclotron (IBA Cyclone 18/9, IBA, Louvain-la-Neuve, Belgium) by irradiation of H_2_^18^O with 18-MeV protons. Female Wistar rats (Janvier labs, Le Genest-Saint-Isle, France) were housed in individually ventilated cages in a thermoregulated (22 °C) and humidity-controlled environment under a 12 h/12 h day/night cycle with free access to food and water. Animal experiments were conducted according to the Belgian code of practice for the care and the use of animals, after approval from the university animal ethics committee.

### Radiosynthesis

**Fig. 2:**
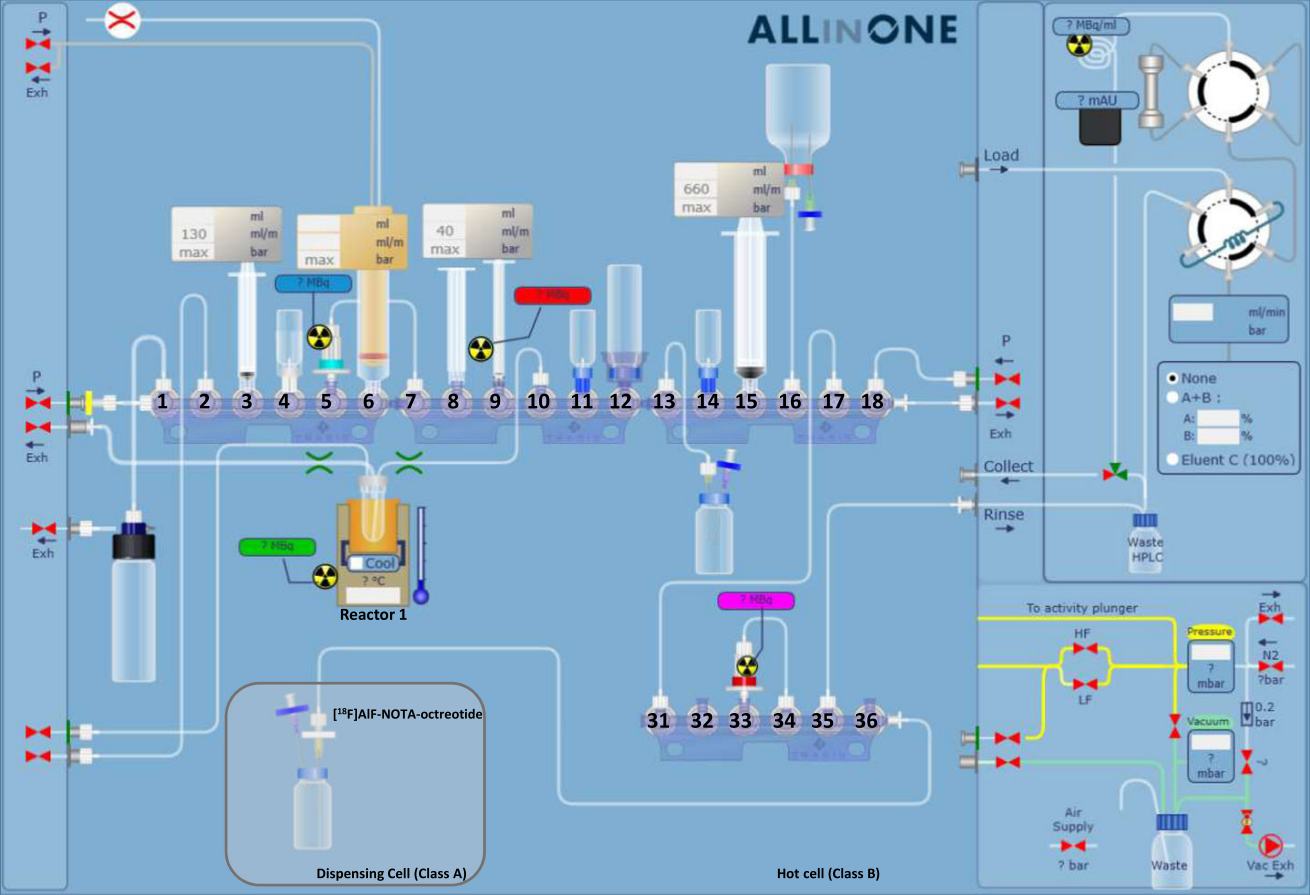
Trasis AllinOne® radio-synthesizer platform: layout of the disposable cassette used for the production of [^18^F]AlF-NOTA-octreotide

[^18^F]AlF-NOTA-octreotide was synthesized in an AllInOne® synthesis module (Trasis, Ans, Belgium). The layout of the cassette is presented in Fig. 2 and the positioning of reagents and materials is depicted in Table 1. During the placement of vials and reagents on the cassette, the 4 mL cyclic olefin copolymer reactor was prefilled with 25 μL of 2 mM aluminum chloride (AlCl_3,_ anhydrous, powder, 99.999% trace metals basis, Sigma-Aldrich) in sodium acetate buffer (0.1 M, pH 4.1).

**Table 1:** Positioning of reagents and materials for the production of [^18^F]AlF-NOTA-octreotide on the Trasis AllinOne® radio-synthesizer platform

Manifold position	Reagents or materials
1 *vertical*	Silicone tubing to [^18^O]H_2_O recovery vial
1 *horizontal*	Silicone tubing to pressure inlet
2	Silicone tubing to exhaust
3	Syringe of 3 ml (S1)
4	QMA eluent
5	QMA cartridge
6	H_2_^18^O/^18^F inlet reservoir (S2)
7	Silicone tubing to QMA cartridge at position 5
8	[^18^F] NaF reservoir
9	Syringe of 1 mL (S3)
10	Silicone tubing to reactor
11	NOTA-octreotide precursor
12	HPCE grade water
13	Silicon tubing to dilution vial
14	EtOH
15	Syringe of 20 mL (S5)
16	Formulation solution
17	Silicone tubing to position 31
18 *vertical*	Silicone tubing to pressure inlet
18 *horizontal*	Silicone tubing to exhaust
31	Silicone tubing from position 17
33	C18 cartridge
34	Silicone tubing to C18 cartridge at position 33
35	Silicone tubing to waste
36 *horizontal*	Silicone tubing to dispensing cell

[^18^F] fluoride (54.0 ± 10.8 GBq) was transferred to the module and trapped on a Sep-Pak light Accel plus anion exchange cartridge (Cl^−^ form: Waters, Milford, Massachusetts, USA). The cartridge was washed with 6 mL of water (HPCE grade, Sigma Aldrich). [^18^F] fluoride was eluted from the QMA cartridge into a reservoir (5 mL Inject syringe; BBraun, Melsungen, Germany) (V8) with 500 μL of the eluent solution consisting out of 250 μL NaCl 0.9% (99.999% trace metals basis NaCl (Sigma Aldrich) in HPCE grade water (Sigma Aldrich)) and 250 μL absolute ethanol. 250 μL of the [^18^F] fluoride containing eluate was transferred to the reactor (V9) containing the AlCl_3_ solution. The solution was stirred for 2 min at room temperature under gentle nitrogen flow (N_2_) to form [^18^F]AlF.

The precursor solution (600 μL of 0.2 mg/mL NOTA-octreotide and 0.95 mg/mL sodium ascorbate in sodium acetate 0.1 M pH 4.1/ absolute ethanol (50/50 V/V)) was added to the reactor which was sealed and heated for 10 min at 100 °C. Around 100 μl of precursor solution is left in the precursor vial (vial 11) after transfer to the reactor. Next, the reactor was cooled to 40 °C and the reaction mixture was transferred to a dilution vial (V13) filled with 15 mL formulation solution (EtOH/NaAsc 0.59% in NaCl 0.9% 1.1/11.9 V/V in water for injection), and mixed under gentle nitrogen flow (N_2_). The diluted solution was transferred over a Sep-Pak light C18 cartridge (Waters) which was preconditioned with 5 mL absolute EtOH and 10 mL water.

Afterwards, the cartridge was washed with 20 mL formulation solution and flushed with nitrogen to remove free [^18^F] fluoride and unreacted [^18^F]AlF. [^18^F]AlF-NOTA-octreotide was eluted from the SPE cartridge to the dispensing cell with 1.6 mL absolute EtOH and the SPE cartridge was flushed with 17.4 mL of the formulation solution. The eluate was passed through a 0.22 μm sterile filter (Millex-GV, 0,22 μm, PVDF, 13 mm, Merck KGaA, Darmstadt, Germany) into a sterile 25 mL dose vial. The final drug product solution ([^18^F]AlF-NOTA-octreotide in EtOH/NaAsc 0.59% in NaCl 0.9% in water for injection) was measured in an ionization chamber-based activity meter (COMECER VIK-203, Comecer S.p.A., Castel Bolognese, Italy) and samples were taken for quality control.

### Validation of analytical procedures

#### High pressure liquid chromatography (HPLC) method used for the identification, radiochemical purity and chemical purity of the active ingredient and its related compounds in the drug product

A Shimadzu LC20A HPLC System (Shimadzu, Kyoto, Japan) coupled in series to a DAD-UV detector (wavelength = 220 nm) (Shimadzu, Kyoto, Japan) and a shielded 3-in. NaI (Tl) scintillation detector connected to a single channel analyzer (Gabi box, Elysia-Raytest, Straubenhardt, Germany) was used. The system was equipped with a Waters XBridge® C18 column (3.5 μm, 3.0 × 100 mm) eluted with a gradient at a flowrate of 0.8 mL/min with mixtures of mobile phase A: ammonium acetate 0.05 M pH 5.5 and acetonitrile as mobile phase B. The elution gradient is shown in Table 2.

**Table 2:** Elution gradient HPLC method. *Mobile phase A (ammonium acetate 0.05 M pH 5.5), mobile phase B (acetonitrile)*

Time (min)	%A	%B
0–5	95	5
5.1–35	80 → 75	20 → 25
35.1–40	95	5

**Fig. 3:**
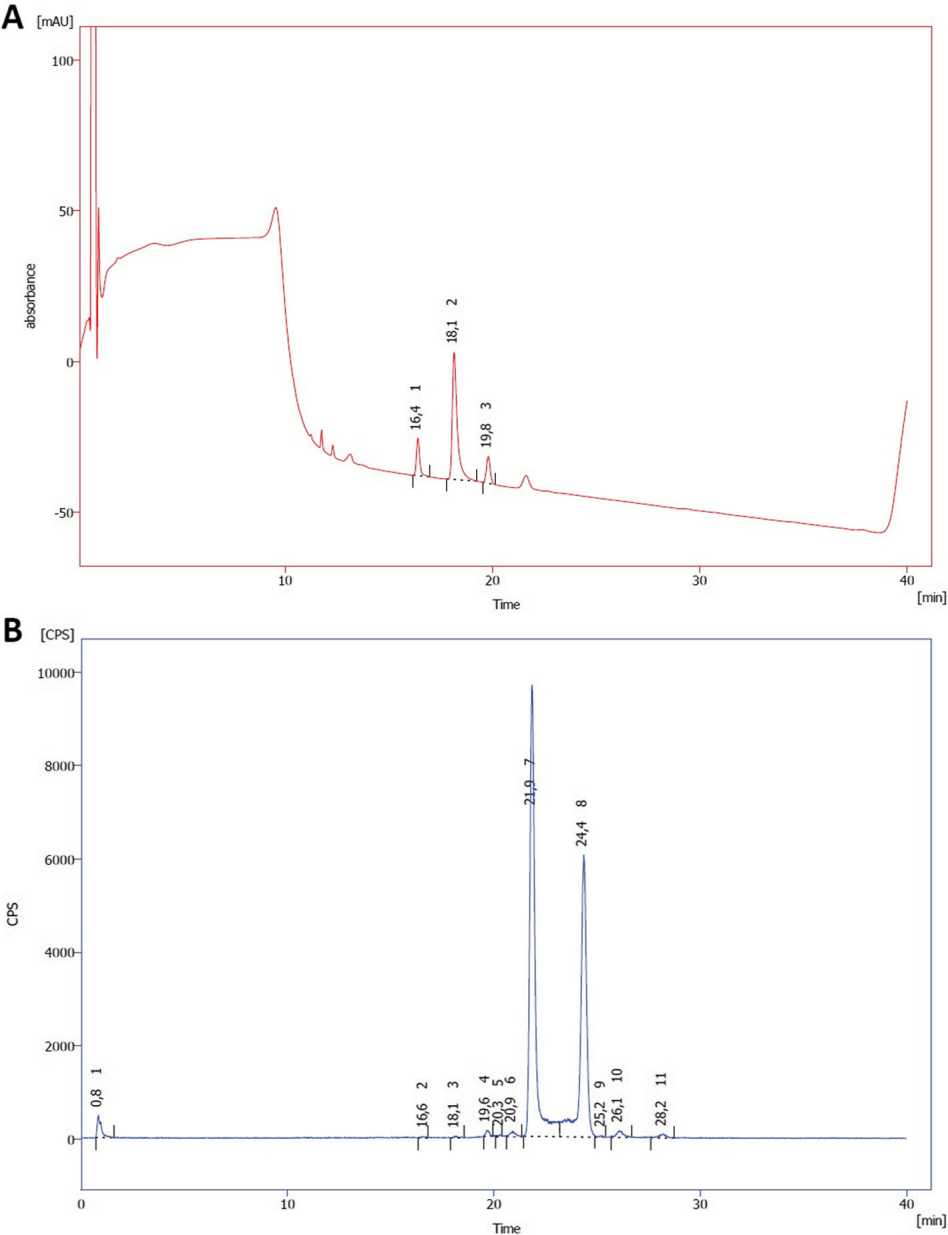
Representative chromatogram (220 nm) of the reference solution (A) and radiochromatogram of [^18^F]AlF-NOTA-octreotide at the end of synthesis (**b**). **a**) Peak 1 (16.4 min) corresponds to NOTA-octreotide, peak 2 (18.1 min) corresponds to octreotide and peak 3 (19.8 min) corresponds to Fe-NOTA-octreotide. **b**) [^18^F]AlF-NOTA-octreotide elutes as two stereoisomers (Peak 7: 21.9 min and Peak 8: 24.4 min), Peak 1 (0.8 min) corresponds to free ^18^F/Al^18^F and Peaks 2–6 and 9–11 correspond to radiolytic degradation products of [^18^F]AlF-NOTA-octreotide

The HPLC method was validated for specificity, linearity and method precision. Quantification and detection limits (LOQ and LOD) of NOTA-octreotide, AlF-NOTA-octreotide and metal complexes of NOTA-octreotide were determined using the UV response factor for NOTA-octreotide (Fig. 3). Prior to the analysis of a batch (20 μL of the [^18^F]AlF-NOTA-octreotide drug product solution), the HPLC analysis system suitability was validated by injection of 20 μL of a reference solution (10 μg/mL NOTA-octreotide trifluoroacetate and 20 μg/mL octreotide acetate in formulation solution (EtOH/NaAsc 0.59% in NaCl 0.9% 1.1/11.9 V/V in water for injection)) followed by a 20 μL blank analysis (formulation solution) (Additional file 1: Figure S1).

The recovery of [^18^F]F^−^, [^18^F] AlF and [^18^F]AlF-NOTA-octreotide from the HPLC method was determined. [^18^F] AlF was prepared by adding 25 μL of 2 mM aluminum chloride (AlCl_3,_ anhydrous, powder, 99.999% trace metals basis, Sigma-Aldrich) in sodium acetate buffer (0.1 M, pH 4.1) to 100 μL of an aqueous solution [^18^F] fluoride (37 MBq/mL). After 3 min, 875 μL formulation solution (EtOH/NaAsc 0.59% in NaCl 0.9% 1.1/11.9 V/V in water for injection) was added. After injection of 20 μL of [^18^F]F^−^, [^18^F] AlF or [^18^F]AlF-NOTA-octreotide in formulation buffer (3.7 MBq/mL, EtOH/NaAsc 0.59% in NaCl 0.9% in water for injection), elution was started with or without an HPLC column in the flow path and the eluate was collected in a tarred tube. The elute was mixed and the radioactivity of a sample of 0.5 g was counted. The data from the gammacounter were used to calculate the recovery (as percentage of [^18^F]F^−^, [^18^F] AlF or [^18^F]AlF-NOTA-octreotide recovery, namely = counts per minute for eluate with column × 100 divided by the counts per minute for eluate without column).

#### Determination of residual solvents by gas chromatography (GC)

Residual solvents were determined by gas chromatography analyses on a Shimadzu GC-20i0 plus (Kyoto, Japan) gas chromatograph controlled by LabSolutions software, coupled to a Shimadzu AOC-20i (Kyoto, Japan) auto-injector and Shimadzu AOC 20s auto-sampler. The GC analysis was performed using a Supelco Equity™ column (5 μm film thickness, 0.32 mm diameter and 30 m length) with a gradient mode (temperature gradient: 0–5 min: 40 °C; 5–12 min: linear gradient from 40 °C to 200 °C; 12–15 min: 200 °C) at a linear velocity of 40 cm/s. The GC method used for the determination of residual solvents in the drug product is validated for specificity and linearity. Method precision was determined by 10 replicate injections of standards of methanol (1%), acetone (1%), tetrahydrofuran (1%), DMF (1%), DMSO (1%), t-amylOH (1%), EtOH (1%) and CH_3_CN (1%). The detection and quantification limits of ethanol were determined (LOD EtOH: 0.0006%; LOQ EtOH: 0.0020%).

### Quality control

The acceptance criteria for the drug product are given in Table 3. ([^18^F]AlF-NOTA-octreotide is obtained as a mixture of two stereoisomers, see Fig. 3).

**Table 3:** Tests parameters, acceptance criteria and test methods applied for [^18^F]AlF-NOTA-octreotide

Test Parameters	Acceptance Criteria	Test Methods
1. Identification	Stereoisomer 1 has a relative retention of 1.3 (±10%) with reference to NOTA-octreotide	HPLC with radiodetector and UV/VIS detector
	Stereoisomer 2 has a relative retention of 1.5 (±10%) with reference to NOTA-octreotide	
2. Radiochemical purity		Radio-HPLC
a. [^18^F]AlF-NOTA-octreotide	≥91% of total radioactivity	
b. Sum [^18^F]F^−^ and [^18^F]AlF	≤5%	
Chemical purity		HPLC with UV/VIS detector
a. Amount (μg) of AlF-NOTA-octreotide, NOTA-octreotide and metal complexes of NOTA-octreotide in total volume to be injected^a^	≤50 μg per injected volume	
b. Amount (μg) of sum of unidentified chemical impurities in total volume to be injected^a^	≤50 μg per injected volume	
4. pH	pH of the finished product is 4.5–8.5	pH strip
5. Integrity of the sterile filter membrane	Bubble point ≥3.4 bar	Bubble point determination
6. Appearance	Colourless and particle-free	Visual inspection
7. Residual solvent		GC
a. EtOH	≤10%v/v	
b. Residual solvent	Conforms Ph Eur.	
8. Total Radioactivity	200–12,110 MBq/batch^c^	Dose calibrator
9. Radionuclide identity- approximate half-life (T_1/2_)	T_1/2_ = 105–115 min	Two time point radioactivity measurement in dose calibrator
10. Radionuclide identity – gamma spectrometry	Gamma energy is 501–521 keV	Gamma spectrum on NaI (Tl) spectrometer
11. Radionuclide purity	≤0.1% of the activity of fluorine-18	Gamma spectrum on NaI (Tl) spectrometer
12. Sterility	No growth after 14 days incubation at 37 °C^b^	Current Ph. Eur. < 2.6.1>
13. Bacterial Endotoxins	≤175 IU per injected volume	LAL-test Current Ph. Eur. < 2.6.14>

^a^*Calculated using the UV response factor for NOTA-octreotide*

^b^*Conform Ph. Eur*

#### Identification

The identity of [^18^F]AlF-NOTA-octreotide is confirmed using the validated radio-HPLC method by determining the relative retention of the principal peaks (1 and 2) in the radiochromatogram relative to the NOTA-octreotide peak obtained with the reference solution using the UV/VIS detector.

#### Radiochemical purity

The radiochemical purity of [^18^F]AlF-NOTA-octreotide is determined using the validated radio-HPLC method by integration of the principal peaks (1 and 2) in the radiochromatogram. The fraction of free [^18^F]F^−^ or [^18^F] AlF is determined by integration of the peaks in the radiochromatogram with a relative retention time between 0 and 0.2 with reference to the NOTA-octreotide peak obtained with the reference solution using the UV/VIS detector.

#### Chemical purity

The chemical purity of [^18^F]AlF-NOTA-octreotide drug product is determined using the validated HPLC method. The amount of AlF-NOTA-octreotide, NOTA-octreotide and metal complexes of NOTA-octreotide in the total volume to be injected is determined by integration of the peaks in the UV chromatogram (wavelength = 220 nm) with a relative retention time between 0.6 and 1.6 with reference to the NOTA-octreotide peak obtained with the reference solution using the UV/VIS detector, by subtracting peaks observed in the blank analysis and by using the UV response factor for NOTA-octreotide. The amount of the sum of unidentified chemical impurities in total volume to be injected is determined by integration of the peaks in the UV chromatogram with a relative retention time between 0.2 and 0.6 and between 1.6 and 2 with reference to the NOTA-octreotide peak obtained with the reference solution using the UV/VIS detector, by subtracting peaks observed in the blank analysis and by using the UV response factor for NOTA-octreotide.

#### pH

pH values were measured with a pH strip (MQuant® pH 0–14, Merck KGaA Darmstadt, Germany).

#### Integrity of the sterile filter membrane

The integrity of the sterile filter membrane was determined using the bubble point test.

#### Radionuclide identity: determination of physical half-life and gammaspectrum

The radionuclide identity was determined by half-life calculation of the drug product. A gamma spectrum was recorded using a gamma spectrometer (Elisya-Raytest, Mucha, GinaStar6 software).

#### Sterility

The drug product was tested for microbiological contamination by direct inoculation in fluid thioglycolate medium and soya-casein digest medium incubation according to Ph. Eur. < 2.6.1>. Environmental monitoring of the dispensing cell (GMP class A) was controlled using contact plates (Tr. Soy Cont. A. +LT -ICR+ and TSA w. LTHTh - ICR 30 mL, Merck KGaA Darmstadt, Germany). Presence of particles in the dispensing cell was monitored with a microbial impactor (BioCapt® Single-Use Microbial Impactor, Particle measuring system, Boulder, Colorado, USA).

#### Bacterial endotoxins

Endotoxins were quantified in the drug product solution using a portable LAL test system (Endosafe PTS, Charles-River, Wilmington, USA) according to Ph. Eur. < 2.6.14 > .

#### Radionuclidical purity: gamma spectrum of decayed sample

The radionuclide purity was determined by gamma-spectrometry (Elisya-Raytest, Mucha, GinaStar6 software) after decay of fluorine-18 (3–7 days after end of production).

#### Radio-LC-HRMS analysis to confirm identity

A Dionex Ultimate 3000 UHPLC System (Thermo Fisher Scientific, Sunnyvale, USA) coupled in series to a UV detector, a 3-in. NaI (Tl) radioactivity detector, and an ultra-high resolution time-of-flight mass spectrometer with electron spray ionization (ESI) (MaXis Impact, Bruker, Bremen, Germany) was used.

The system was equipped with a Waters Acquity UPLC BEH C18 column (1.7 μm 2.1 X 50 mm) using a gradient at a flowrate of 0.6 mL/min with mobile phase A: H_2_O, 0.1% HCOOH and mobile phase B: acetonitrile, 0.1% HCOOH. The column was heated at 40 °C. The elution gradient was: 0–2 min: 95% A; 2–8 min: from 95% A to 5% A; 8–10 min: 5% A; 10–12 min.: from 95% A to 5% A. UV monitoring of the eluate was performed at 220 nm. Calculated molecular ion mass values were obtained using Compass Isotope Pattern (version 3.2, Bruker) software.

#### Stability tests

The stability of [^18^F]AlF-NOTA-octreotide was determined using the validated radio-HPLC method by integration of the principal peaks (1 and 2) in the radiochromatogram. Stability tests were performed on the drug product at different radioactivity concentrations at room temperature up to 6 h after preparation.

**Table 4:** Gradient used for radiometabolite analysis *Mobile phase A (ammonium acetate 0.05 M pH 5.5), mobile phase B (acetonitrile)*

Time (minutes)	A %	B %	Flowrate (mL/min)
0	99	1	0.5
4	99	1	0.5
4.1	99	1	1
14	10	90	1
17	10	90	1
17.1	10	90	0.5
25	99	1	0.5

### Preclinical studies

#### Radiometabolite analysis in rats

Female Wistar Rats (*n* = 3) were injected with [^18^F]AlF-NOTA-octreotide (1 ml, 74 MBq, 40 nmoles) via a tail vein under anesthesia (2.5% isoflurane in O_2_ at 1 L/min flow rate) and kept under anesthesia during the experiment. Urine samples (30 min post-injection) were collected and filtered through a 0.22 μm filter (Millex-GV, 0,22 μm, PVDF, 13 mm, Merck KGaA, Darmstadt, Germany) and stored on ice. Blood samples (10 and 30 min post-injection) were collected via a tail vein in EDTA containing tubes (0.5 mL, K_2_EDTA MiniCollect tubes; Greiner Bio-One) and stored on ice. The tubes were centrifuged for 10 min at 2333 x g, plasma was collected and the recovery (> 95%) was determined using a gammacounter (Perkin-Elmer, Wizard^2^ 2480, Waltham, Massachusetts, USA). As a control, urine and blood samples from non-injected animals were spiked with [^18^F]AlF-NOTA-octreotide (0.2–1 MBq) and processed using the same procedure. 20 μL of the plasma or urine sample was injected on a Hitachi Elite Lachrom HPLC System (Tokyo, Japan) coupled in series to a shielded 3-in. NaI (Tl) scintillation detector connected to a single channel analyser (Gabi box, Elysia-Raytest) equipped with a Chromolith performance column (C_18_, 4.6 mm × 100 mm, Merck KGaA, Darmstadt, Germany). The elution gradient is shown in Table 4 with mobile phase A: ammonium acetate 0.05 M pH 5.5 and acetonitrile as mobile phase B. The eluate was collected in fractions of 1 mL using an automated fraction collector (Bio Rad model 2110, Hercules, California, USA) and radioactivity was measured using the gamma counter. The recovery of the injected radioactivity was > 95% (sum counts per minute of collected fractions × 100 divided by the counts per minute of the injected sample).

### In vivo pharmacokinetics and biodistribution

Dynamic PET scans were performed on a small animal PET/MR scanner consisting of a Biospec 70/30 small animal MRI scanner (30 cm horizontal bore, actively shielded gradients of 200 mT m^− 1^, Bruker Biospin, Ettlingen, Germany) with an Albira PET Insert (three rings of monolythic LYSO crystals coupled to silicon photomultipliers; Bruker Biospin). Female Wistar Rats (210–282 g) were anaesthetized with a 5% isoflurane/oxygen mixture before being maintained at 1–2% isoflurane/1 L/minute throughout the experiment. Rats tail veins were cannulated before being placed in the imaging cell (Bruker Biospin). Temperature and respiration were monitored throughout the imaging procedure using a physiological monitoring system (SA Instruments, Stony Brook, NY, USA). At the start of scanning, rats were injected with [^18^F]AlF-NOTA-octreotide (7.8–26.8 MBq, apparent molar activity 25 ± 7 GBq/μmol) in presence or absence of octreotide acetate (2.5 mg/kg). Blocking scans were performed the following day in the same animals. Dynamic scans (with pituitary to kidneys in the PET FOV) were carried out for 75 min, during which respiration-gated MRI acquisitions were made. Two static PET scans and 3D MRI scans were simultaneous acquired at 100–120 and 120–140 min p.i. using two animal bed positions with 20% overlap to provide whole body coverage. MR images were acquired with a quadrature radio-frequency resonator (transmit/receive, 72 mm diameter, Bruker Biospin). After the acquisition of localizer scans, the following 3D MR scans were obtained for the two bed positions: 3D spin-echo MRI (RARE sequence, TR = 500 m seconds (ms), TE = 18.8 ms, RARE factor = 8, FOV 14x6x6 cm, isotropic resolution of 0.5 mm) and 3D gradient-echo MRI (FLASH sequence, TR/TE = 12.3/ 2.3 ms, FOV 14x6x6cm, isotropic resolution of 0.5 mm)). Dynamic PET data were divided into timeframes (4x15s, 1x240s, 7x600s) and reconstructed using an MLEM algorithm with 24 iterations and an isotropic voxel size of 0.5 mm, with corrections for randoms, scatter and deadtime. Static data were reconstructed as a single timeframe using the same reconstruction parameters. PET images were normalized to injected activity and animal weight to give standardized uptake values (SUVmean), and selected organs were outlined to create volumes of interest after coregistration with MR images. All image analysis was carried out using the PFUSIT module of PMOD (v 4.004, PMOD Technologies Ltd., Zurich, Switzerland). Quantitative data are expressed as mean ± standard error of the mean (SEM).

### Statistical analysis

Quantitative data are expressed as mean ± standard deviation (SD) unless stated otherwise. Means were compared using a paired single-tailed Student t-test. Values were considered statistically significant for *P* < 0.05.

## Results

### Radiochemistry

The production process of [^18^F]AlF-NOTA-octreotide has been validated by three validation production runs using an identical production protocol, resulting in three batches of [^18^F]AlF-NOTA-octreotide (batch A-C). [^18^F]AlF-NOTA-octreotide was produced with a final batch activity of 10.8 ± 1.3 GBq at end of synthesis (EOS) with a radiochemical yield of 26.1 ± 3.6% (dc), calculated from the starting activity of [^18^F]F^−^ in the target. The total synthesis time was 40 ± 3 min, starting from the end of [^18^F]F^−^ transfer to the synthesis module, to obtain the purified drug product [^18^F]AlF-NOTA-octreotide.

### Validation of analytical procedures

The HPLC method used for the identification and quantitative determination of the active ingredient and its related compounds in the drug product was validated for the parameters presented in Table 5.

**Table 5:** Method validation summary for HPLC and GC method

Validation parameters	Results
HPLC method	
Specificity	The method is specific for [^18^F]AlF-NOTA-octreotide, NOTA-octreotide, metal complexes of NOTA-octreotide and octreotide (resolution ≥1.5)
Linearity	The method is linear in the tested range Range 2.01–29.92 μg/mL, R^2^ > 0.995 for NOTA-octreotide
Method precision	6 repeated injections of 20 μL of a 5.026 μg/mL NOTA-octreotide solution yield a % RSD AUC of 3.5% and a % RSD of retention time of 0.2%.
Quantification and detection limits of NOTA-octreotide, AlF-NOTA-octreotide and metal complexes of NOTA-octreotide^a^	LOD = 0.30 μg/mL
	LOQ = 1.0 μg/mL
Recovery [^18^F]F^−^, [^18^F] AlF and [^18^F]AlF-NOTA-octreotide	99.0 ± 1.4%, 100.1 ± 1.1% and 101.9 ± 1.8%
GC method	
Specificity	The method is specific for EtOH
Linearity	R^2^ > 0.99 for all tested solvents, range: 0.005% to 5% for CH_3_CN and DMSO; 0.005% to 10% for EtOH
Method precision	The % RSD of the AUC was < 5% and the %RSD of the retention time was < 1% for all tested solvents.
Quantification and detection limits	LOD EtOH: 0.0006%; LOQ EtOH: 0.0020%

^a^Calculated using the UV response factor for NOTA-octreotide

Injection of the reference solution (10 μg/mL NOTA-octreotide trifluoroacetate and 20 μg/mL octreotide acetate) on the HPLC system is used as a system suitability test before batch analysis. A resolution of at least 1.5, between NOTA-octreotide and octreotide, is required. The proposed HPLC method allows efficient separation between NOTA-octreotide and octreotide (R_s_: 4.02 ± 0.07). A chromatogram of the reference solution is shown in Fig. 3a. Peak 1 (16.4 min) corresponds to NOTA-octreotide, peak 2 (18.1 min) corresponds to octreotide and interestingly, a third peak was observed eluting at 19.8 min which was later found to correspond to Fe-NOTA-octreotide as determined by LC-MS. Further, the system is specific for [^18^F]AlF-NOTA-octreotide (eluting as two stereoisomers at 21.9 and 24.4 min, respectively) which is efficiently separated from NOTA-octreotide and octreotide. A representative radiochromatogram of [^18^F]AlF-NOTA-octreotide is shown in Fig. 3b. Residual solvents in the drug product are quantified using a validated GC method (Table 5).

### Quality control

Validation runs have been performed (batch A-C) and the batch details and results are shown in Table 6. The identity of [^18^F]AlF-NOTA-octreotide was confirmed by analyzing the relative retention of the principal peaks (1 and 2) in the radiochromatogram with reference to the NOTA-octreotide peak obtained with the reference solution using the UV/VIS detector.

**Table 6:** [^18^F]AlF-NOTA-octreotide validation runs batch analysis

Test	Specification	Batch A	Batch B	Batch C
1. Identification	Stereoisomer 1 has a relative retention of 1.3 (±10%) with reference to NOTA-octreotide	Conforms	Conforms	Conforms
		Relative retention stereoisomer 1: 1.3	Relative retention stereoisomer 1: 1.3	Relative retention stereoisomer 1: 1.4
	Stereoisomer 2 has a relative retention of 1.5 (±10%) with reference to NOTA-octreotide			
		Relative retention stereoisomer 2: 1.5	Relative retention stereoisomer 2: 1.5	Relative retention stereoisomer 2: 1.5
2. Radiochemical purity				
a. [^18^F]AlF-NOTA-octreotide	≥91% of total radioactivity	96.5%	96.3%	96.0%
b. [^18^F]F^−^ or [^18^F]AlF	≤5%	0.4%	1%	0.5%
3. Chemical purity				
a. Amount (μg) of AlF-NOTA-octreotide, NOTA-octreotide and metal complexes of NOTA-octreotide in total volume to be injected^c^	≤50 μg per injected volume^a^	162 μg/18.6 ml	94 μg/18.9 ml	63 μg/18.2 ml
b. Amount (μg) of sum of unidentified chemical impurities in total volume to be injected^c^	≤50 μg per injected volume	<LOD	<LOD	<LOD
4. pH	pH of the finished product is 4.5–8.5	7	7	7
5. Integrity of the sterile filter membrane	Bubble point ≥3.4 bar	3.6 bar	3.8 bar	bar
6. Appearance	Colorless and free particle matter	Conforms	Conforms	Conforms
7. Residual solvent				
a. EtOH	5–10% v/v	10% v/v	10% v/v	9% v/v
b. Residual solvents	Conforms Ph Eur.	Conforms	Conforms	Conforms
8. Total Radioactivity	200–12,110 MBq/batch	9542 MBq	12,110 MBq	10,660 MBq
9. Radionuclide identity- approximate half-life (T_1/2_)	T_1/2_ = 105–115 min	106.2 min	107.8 min	111.6 min
10. Radionuclide identity – gamma spectrometry	Gamma energy is 501–521 keV	518 keV	516 keV	516 keV
11. Sterility	No growth after 14 days incubation at 37 °C conform Ph. Eur.	Conforms	Conforms	Conforms
12. Bacterial Endotoxins	≤175 IU per injected volume	Conforms	Conforms	Conforms

^a^Calculated using the UV response factor for NOTA-octreotide LOD = Limit of Detection

The radiochemical purity of [^18^F]AlF-NOTA-octreotide was 96.3 ± 0.2% with 0.6 ± 0.3% free [^18^F]F^−^ or [^18^F]AlF. The apparent molar activity, based on the amount of AlF-NOTA-octreotide, NOTA-octreotide and metal complexes of NOTA-octreotide, was found to be 160 ± 75 GBq/μmol. The pH of the finished product was found to be consistently 7 and the integrity of the sterile filter was confirmed using a bubble point test (≥3.4 bar). The percentage of ethanol was found to be 9.7 ± 0.5% and other residual solvents were below the limits of the Ph. Eur (chapter 5.4) and ICH Q3C (R7) implemented by the European medicinal agency (EMA/CHMP/ICH/82260/2006 2018). The radionuclide identity (Fluorine-18) was confirmed using gamma spectrometry and with the approximate half-life determination test. All three batches were found to be sterile and were complying with Ph. Eur. requirements for bacterial endotoxins (Ph. Eur. < 2.6.14>).

Radio-LC/HRMS additionally confirmed the identity of [^18^F]AlF-NOTA-octreotide (performed on batch B). The peak in the base peak chromatogram, co-eluting with the radioactive signal, was identified as AlF-NOTA-octreotide eluting as two stereoisomers. (observed m/z = 674.7772, z = 2, theoretical molecular ion mass C_61_H_83_N_13_O_15_S_2_AlF 674.7759 ([MH2]^2+^, z = 2). We also observed the presence of the aluminum complex of NOTA-octreotide (m/z = 1328.5400 ([M]^+^, z = 1). The theoretical molecular mass calculated for C_61_H_83_N_13_O_15_S_2_Al is 1328.5383 ([M]^+^, z = 1).

The stability of [^18^F]AlF-NOTA-octreotide in the formulation solution was determined using the validated radio-HPLC method. At a maximum concentration of 640 MBq/mL (12,110 MBq/18.9 mL), low levels of byproducts from radiolysis were observed and the radiochemical purity remained > 96% over a time period of 6 h for the three validation production batches. Batch C was additionally analyzed after 17 h and no change of the radiochemical purity was observed as shown in Table 7.

### Preclinical studies

#### Radiometabolite analysis in rats

**Fig. 4:**
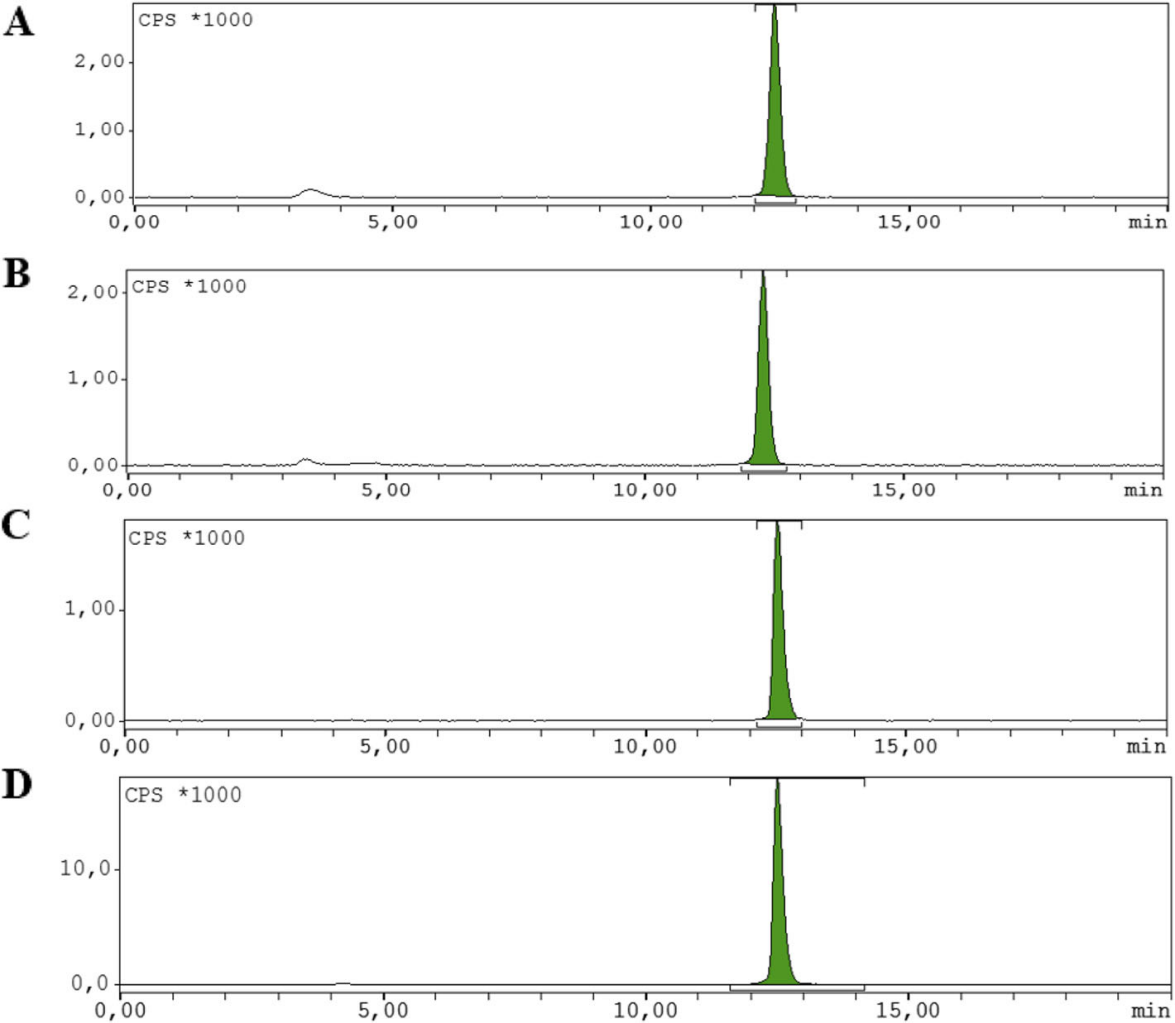
Radiometabolite analysis in plasma and urine **a**) Radiochromatogram of rat plasma sample spiked with [^18^F]AlF-NOTA-octreotide; **b**) Radiochromatogram of rat urine sample spiked with [^18^F]AlF-NOTA-octreotide **c**) Radiochromatogram of plasma sample 10 min. Post-injection; **d**) Radiochromatogram of urine sample 30 min. Post-injection;. More than 98% of fluorine-18 corresponded to the parent tracer indicating its high in vivo stability

The in vivo metabolic stability of [^18^F]AlF-NOTA-octreotide was studied in rats by analyzing plasma (10 and 30 min post-injection) and urine (30 min post-injection). Both in plasma and in urine samples more than 98% of fluorine-18 was present as the parent compound (Fig. 4).

#### In vivo biodistribution

**Fig. 5:**
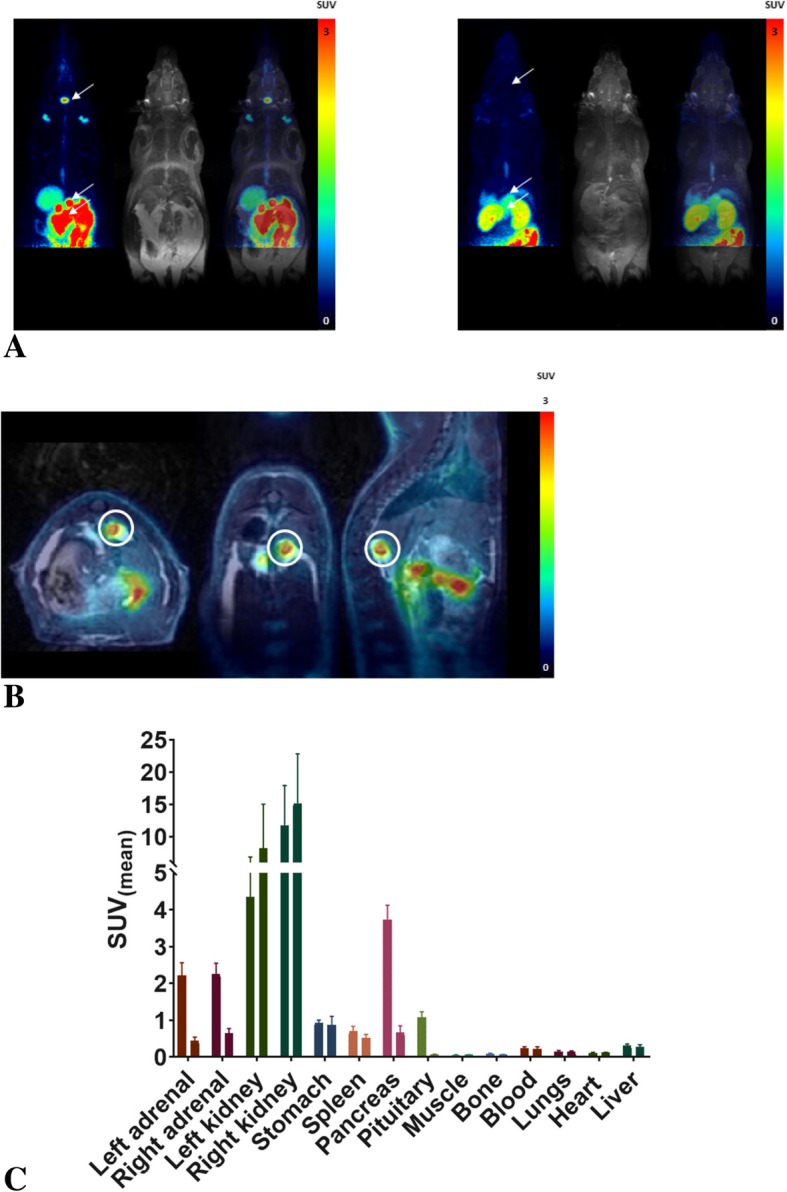
In vivo biodistribution of [^18^F]AlF-NOTA-octreotide in control and blocking (co-injection with 2.5 mg/kg octreotide). **a**) Maximum intensity projections of PET, MRI and PET/MRI fusion data at 60–70 min post-injection, with SSTR2-expressing organs (pituitary and adrenal glands, pancreas) highlighted by arrows in representative naïve (left) and blocked (right) animals. MR images were acquired in two bed positions with at least 20% overlap. Images shown above were generated after fusion of both images. **b**) Single slice image at 60–70 min post-injection centered on the right adrenal gland (circled in white) to illustrate MR-guided organ delineation. **c**) Organ concentration of [^18^F]AlF-NOTA-octreotide at 2 h post injection for selected organs (*n* = 3) in control (left) and blocking (right) conditions

The uptake of [^18^F]AlF-NOTA-octreotide in a range of organs as determined via μPET-MR imaging is summarized in Fig. 5 and Additional file 1: Figure S3. High uptake was seen in SSTR2-expressing organs which was significantly (*P* < 0.05) reduced upon co-injection of 2.5 mg/kg octreotide (adrenals SUV 2.23 ± 0.22 vs 0.54 ± 0.07, pituitary SUV 1.07 ± 0.16 vs 0.07 ± 0.01, pancreas SUV 3.74 ± 0.39 vs 0.66 ± 0.16) at 2 h post-injection respectively. Tracer concentration in blood and other organs was generally low (SUV < 0.6) at 2 h post-injection and only limited defluorination was observed (bone SUV 0.45 ± 0.03 at 2 h post-injection in naïve animals).

## Discussion

### Radiochemistry

Common radiofluorination procedures used for small organic molecules, applying organic solvents and high temperature, are often not suitable for complex peptides or biomolecules sensitive to these harsh reaction conditions. Therefore, many indirect approaches with ^18^F-labeled synthons have been described (Olberg et al. 2010). A multistep process is time-consuming and complicates automation and GMP compliant production. The development of the Al^18^F-method by McBride et al. opened new possibilities for the direct radiolabeling of peptides and proteins with the radionuclide of choice, fluorine-18. Laverman et al. successfully applied the Al^18^F-method for the production of [^18^F]AlF-NOTA-octreotide (Laverman et al. 2012).

Cost efficient clinical routine production of PET-radiopharmaceuticals requires high batch activity, allowing to inject multiple patients from one batch thus reducing the production cost per patient dose. However, reported radiosynthesis methods for [^18^F]AlF-NOTA-octreotide are mostly manual, starting with low amount of radioactivty and resulting in low batch activity (Laverman et al. 2010; Laverman et al. 2012). Automation allows for high-batch activity GMP compliant production of ^18^F-labeled radiopharmaceuticals, with limited radiation exposure for production operators. Moreover, automation provides higher batch-to-batch reliability thanks to reduction of human factors and tighter control of critical steps during the production process. Recently, automated production of [^18^F]AlF-NOTA-octreotide, on the Trasis AllinOne® synthesizer, was described by Allot L. et al. (Allott et al. 2017). However, further optimization was required to allow GMP production of [^18^F]AlF-NOTA-octreotide for routine clinical use.

Working under metal-free conditions is an important factor for successful Al^18^F-labeling, which is also the case for other chelator-based radiolabeling methods (Šimeček et al. 2013). Several measures were implemented to avoid interference of metals, which reduce radiolabeling efficiency. We used high purity reagents (e.g. trace metals basis AlCl_3_ and NaCl, HPCE grade H_2_O) and buffers used for radiolabeling were chelex-treated to remove trace metal ions. Further, plastic tools and pipettes were used instead of metal weighing equipment and needles. Finally, metal ions may also originate from the cyclotron target, therefore the [^18^F]F^−^ was trapped on Sep-Pak light Accel plus anion exchange cartridge and washed with HPCE grade water before it was used for radiolabeling.

Purification of radiopharmaceuticals is mostly performed using disposable cartridges or by HPLC. Cartridge purification is simple and fast but HPLC purification usually affords better separation. Indeed, HPLC purification allows to separate [^18^F]AlF-NOTA-octreotide from NOTA-octreotide, and from metal complexes of NOTA-octreotide such as Al-NOTA-octreotide and Fe-NOTA-octreotide, resulting in a radiotracer with a high apparent molar activity. Though, the tumor uptake vs A_m_ may be bell shaped when targeting tumors with diagnostic radiopeptides. If too few molecules are administered (high A_m_), a large fraction of the radiotracer can be trapped during its first pass effect by high-affinity low-capacity binding sites in non-target organs such as the liver, and will not reach their target (Vermeulen et al. 2019). Therefore, we opted to perform a cartridge purification instead of an HPLC purification. However, more studies are required to validate these statements with regard to the A_m_ of [^18^F]AlF-NOTA-octreotide for optimal tumor targeting of NETs. This strategy resulted in an apparent molar activity of 160.5 ± 75.3 GBq/μmol, representing a compromise between, e.g., a saturable elimination and receptor saturation (Velikyan et al. 2010). Moreover, SPE cartridge purification and reformulation is easily implementable and reliable on an automated system (Lemaire et al. 1999). As a note, the starting activity of [^18^F]F^−^ was lower during the synthesis of [^18^F]AlF-NOTA-octreotide used for the preclinical studies, resulting in a moderate apparent molar activity (25 ± 7 GBq/μmol) which might have an effect on biodistribution and uptake in SSTR2-expressing organs.

Radiolysis depends on the radioactivity concentration, the A_m_, the chemical structure of the radiopharmaceutical, the solvent and the position of the radiolabel. Radiolysis can occur at any stage during or after production of the radiopharmaceutical. As SPE cartridge purification does not allow to separate [^18^F]AlF-NOTA-octreotide from its radiolysis induced labeled peptide fragments, it is important to prevent radiolysis during the radiolabeling step to achieve a high radiochemical purity.

For this reason, Allott, L*.* et al. recommended to add 45 μL of 9.5 M of ascorbic acid to the reaction mixture (total reaction volume of 1 mL, resulting in a concentration of 75 mg/mL ascorbic acid) to prevent radiolytic degradation of [^18^F]AlF-NOTA-octreotide during the radiolabeling step (Allott et al. 2017). Such a high concentration of ascorbic acid was not needed in our view, as it could cause a drop in pH of the reaction mixture. Indeed, even 50 mg/mL ascorbic acid resulted in a pH drop (pH 3.5) of the reaction mixture, which is below the optimal pH for Al^18^F-radiolabeling of NOTA-octreotide (Laverman et al. 2012). Therefore, we used sodium ascorbate instead of ascorbic acid for subsequent radiolabeling studies as we determined that high concentrations of sodium ascorbate (50 mg/mL) does not significantly change the pH of the reaction mixture. Finally, we only used 0.95 mg/mL sodium ascorbate in the precursor solution, resulting in a final concentration in the reaction mixture of 0.61 mg/mL sodium ascorbate and 50% (V/V) ethanol, which was found to be sufficient to prevent radiolysis. Afterwards, we found that a concentration of 0.61 mg/mL of ascorbic acid also does not alter the pH of the reaction mixture. Therefore, we would advise to use ascorbic acid instead of sodium ascorbate to avoid possible interference of metals, as only ascorbic acid is available in high purity grade (trace metals). The formulation solution containing ethanol and sodium ascorbate effectively prevents radiolysis, as the radiochemical purity remained > 96% over a time period of 6 h.

Since the automated module does not allow to accurately transfer small volumes (25 μL) of AlCl_3_ solution to the reactor, we decided to elute the [^18^F]F^−^ from the QMA cartridge with a mixture of NaCl and ethanol, already containing AlCl_3_. Although this approach initially performed well, we experienced that only a variable fraction of the AlCl_3_ effectively reached the reactor resulting in variable radiochemical yields. Therefore, we decided to add 25 μL of the AlCl_3_ solution directly into the reactor just before the start of the automated synthesis sequence, to avoid any possible loss of AlCl_3_ during transfer.

Finally, critical parameters for efficient Al^18^F-labeling are the ^18 + 19^F^−^-to-Al^3+^ ratio and the chelator-to-Al^3+^ ratio in the labeling reaction mixture (Cleeren et al. 2018). We opted to use only 0.12 mg GMP-grade precursor NOTA-octreotide trifluoroacetic acid precursor per radiolabeling. In order to reduce the amount of fluoride in the reaction mixture, we use only half of [^18^F]F^−^ eluted from the QMA cartridge. Indeed, when we used the whole batch of [^18^F]F^−^ in the reaction mixture, we obtained lower radiochemical yields, formed more radiolysis products and we observed breakthrough of [^18^F]F^−^/[^18^F] AlF during cartridge purification, resulting in lower radiochemical purity.

### Quality control

Before release for clinical use, the PET-tracer is analyzed using quality control methods validated in accordance with the guidelines of ICH Q2(R1) (ICH Q2 (R1) 2005). Further, as [^18^F]AlF-NOTA-octreotide is not described in an individual Ph.Eur. monograph, the requirements described in the general monographs and general texts apply.

RadioHPLC analysis of [^18^F]AlF-NOTA-octreotide using a gradient analysis on a reversed phase column, was described by Laverman et al. using trifluoroacetic acid 0.1% w/v in the mobile phase (pH 2) (Laverman et al. 2012). Ory et al. have shown that [^18^F] fluoride recovery increases with increasing pH for silica-based C18 columns and thus it is recommended to use a mobile phase with a pH higher than 5 (Ory et al. 2015). Consequently, an ammonium acetate buffer 0.05 mM, pH 5.5 was selected as mobile phase resulting in high recovery of [^18^F]F^−^, [^18^F] AlF and [^18^F]AlF-NOTA-octreotide (Table 5). The described gradient system allows efficient detection and quantification of [^18^F]F^−^/[^18^F] AlF, [^18^F]AlF-NOTA-octreotide eluting as two stereoisomers and radiolysis products of [^18^F]AlF-NOTA-octreotide (Fig. 3b).

Laverman et al. already observed the formation of two stereoisomers of [^18^F]AlF-NOTA-octreotide (Laverman et al. 2012) and the isomerization of macrocyclic bifunctional chelator metal complexes was described before (Schlesinger et al. 2011; Tircso et al. 2011; Payne, et al. 2015). Different hypotheses are given to explain isomerization of macrocyclic chelators after metal coordination. One of these could be the formation of two different coordinated complexes or the rotation of carboxylates arms coordinating the metal (Schlesinger et al. 2011). Another hypothesis is that the metal chelation results in conformational difference depending on which side the metal binds to the macrocycle of the bi-functional chelator (Tircso et al. 2011). The fast conformational modification through continuous cycles of metal release and recombination leads to molecules with different physico-chemical properties such as hydrophilicity.

To confirm the fast interconversion of the two isomers, we collected either the first or the second eluting [^18^F]AlF-NOTA-octreotide peak and reinjected the isolated peaks on the same HPLC system. This resulted again in the occurrence of the same two radioactive peaks, suggesting indeed that both [^18^F]AlF-NOTA-octreotide isomers undergo rapid interconversion (Additional file 1: Figure S2). Therefore, no further studies of separate isomers were possible. Interconversion is probably also occurring on the HPLC column itself during analysis as a high baseline signal is observed between the isomer peaks (Fig. 3b). In addition, radio-LC/HRMS analysis showed that both [^18^F]AlF-NOTA-octreotide peaks have an identical molecular ion mass corresponding to the theoretical mass of [^18^F]AlF-NOTA-octreotide.

Injection of a reference solution containing 10 μg/mL NOTA-octreotide trifluoroacetate and 20 μg/mL octreotide acetate in the formulation solution is used as an HPLC system suitability test in analogy to the approach in the Ph. Eur monograph for edotreotide. In our specification used to validate the HPLC system, the minimum resolution value limit for the separation of NOTA-octreotide and octreotide was set to 1.5 (Table 5). The retention time relative to that of NOTA-octreotide is used to determine the identity of [^18^F]AlF-NOTA-octreotide also in analogy with the Ph.Eur. edotreotide monograph where edotreotide and octreotide are used as reference solution for the HPLC system suitability.

In the HPLC chromatogram of the reference solution (Fig. 3a), beside the octreotide and the NOTA-octreotide peaks, an unexpected third peak was observed. The peak was identified as Fe-NOTA-octreotide (C_61_H_82_N_13_O_15_S_2_Fe) after isolation and LC-HRMS analysis (found m/z = 1357.4906 (M + H)^+ 1^, theoretical monoisotopic mass (M + H)^+^ = 1357.4922). Iron is known to form stable complexes with NOTA (Šimeček et al. 2013). Although the reference solution was carefully prepared in metal-free conditions, variable amounts of the peak corresponding to Fe-NOTA-octreotide was observed. In contrast, no Fe-NOTA-octreotide was detected when the reference solution was analyzed directly on the LC-HRMS system, indicating and formation of Fe-NOTA-octreotide in the HPLC system itself with the mobile phase, injection system, column, tubing’s as potential iron sources. A metal-free HPLC system could resolve this problem.

The acceptance criteria depicted in Table 3 for radiochemical purity, endotoxins and sterility are comparable to specifications for other PET-radiopharmaceuticals described in the Ph. Eur. For chemical purity, a limit for the sum of AlF-NOTA-octreotide, NOTA-octreotide and metal complexes of NOTA-octreotide was set to 50 μg based on the monograph of “Gallium (^68^Ga) edotreotide injection” in the Ph. Eur. where specifications are comparable to specifications for edotreotide and metal complexes of edotreotide. For the sum of unidentified chemical impurities, a maximum of 50 μg per injected dose was set, based on the same monograph.

**Table 7:** Stability of [^18^F]AlF-NOTA-octreotide in the formulation buffer

Batch	Radioactivity concentration	Incubation time before reanalysis	Radiochemical purity t = 0	Radiochemical purity after incubation
Batch A	513 MBq/mL	1, 2, 3, 4, 5 and 6 h	96.5%	> 95% after
				1, 2, 3, 4, 5 and 6 h
Batch B	640 MBq/mL	1, 2, 3, 4, 5 and 6 h	96.3%	> 95% after
				1, 2, 3, 4, 5 and 6 h
Batch C	585 MBq/mL	1, 2, 3 and 17 h	96.0%	> 95% after
				1, 2, 3, and 17 h

### Preclinical studies

Biodistribution studies in SSTR2 positive tumor mice showed high specific uptake of [^18^F]AlF-NOTA-octreotide in the tumor and in SSTR2-expressing tissues with little or no in vivo defluorination (Laverman et al. 2012). However, in vivo metabolite studies were lacking. Therefore, we performed plasma and urine radiometabolite studies in healthy rats. Both in plasma and in urine samples more than 98% of fluorine-18 was present as the parent compound [^18^F]AlF-NOTA-octreotide, indicating excellent in vivo stability (Fig. 4). Further, the biodistribution of the radiotracer and the specificity of the binding to the target was confirmed using simultaneous μPET/MRI (Fig. 5), which allowed organ uptake to be delineated in the same animal pre- and post-addition of a blocking dose of octreotide. Uptake was high in SSTR2-expressing organs, and was significantly reduced by co-injection of octreotide (Fig. 5a and Additional file 1: Figure S3). Background tissue and bone uptake was low, resulting in high contrast images and indicating limited defluorination. This set of preclinical results supports translation to clinical evaluation.

Recently, Long et al. published the results of the first human study comparing [^18^F]AlF-NOTA-octreotide to [^18^F] FDG for the detection of NET (Long et al. 2019). High tumor uptake and tumor-to-background ratios were reported. Further, [^18^F]AlF-NOTA-octreotide is being evaluated in a prospective clinical study (NCT03883776) that compares [^18^F]AlF-NOTA-octreotide directly to [^68^Ga]Ga-DOTATATE in NET patients. The first clinical results look promising with no evidence of in vivo defluorination, high SUV_max_ values and high tumor-to-background ratio (TBR) for [^18^F]AlF-NOTA-octreotide (Pauwels et al. 2019).

## Conclusion

^68^Ga-DOTA-peptide PET is the current standard for somatostatin receptor imaging in NET patients, but its batch activity is limited. [^18^F]AlF-NOTA-octreotide is a promising alternative combining the advantages of a chelator-based radiolabeling method with the unique properties of fluorine-18. We developed a robust and automated production process that allows fast and high batch activity production of [^18^F]AlF-NOTA-octreotide allowing centralized production and shipment to remote PET centers. Furthermore, the production process and quality control developed for [^18^F]AlF-NOTA-octreotide is easily implementable in a clinical setting. [^18^F]AlF-NOTA-octreotide showed high in vivo stability and favorable pharmacokinetics with high and specific accumulation in SSTR2 expressing organs which supports clinical translation.

## Supplementary information

**Additional file 1: Figure S1.** Representative chromatogram (220 nm) of the formulation solution (blank). **Figure S2.** Radiochromatogram of [^18^F]AlF-NOTA-octreotide. A) Analytical chromatogram of [^18^F]AlF-NOTA-octreotide at the end of synthesis. **Figure S3.** In vivo biodistribution of [^18^F]AlF-NOTA-octreotide in control and blocking (coinjection with 2.5 mg/kg octreotide)

## Data Availability

All data generated or analyzed during this study are included in this published article [and its supplementary information files].

## Abbreviations

ACN: Acetonitrile
Am: Molar activity
COC: Cyclic olefin copolymer
CT: Computed tomography
dc: Decay corrected
DMF: Dimethyl formamide
DMSO: Dimethyl sulfoxide
EtOH: Ethanol
FLASH: Fast low angle shot
FOV: Field-of-view
GC: Gas chromatography
GMP: Good manufacturing practice
HPCE: High-performance capillary electrophoresis
HPLC: High pressure liquid chromatography
LC: Liquid chromatography
LOD: Limit of detection
LOQ: Limit of quantification
MLEM: Maximum likelihood estimation method
MRI: Magnetic resonance imaging
MS: Mass spectrometry
NaAsc: Sodium ascorbate
NET: Neuroendocrine tumor
PET: Positron emission tomography
PFUSIT: PMOD fuse it tool
RARE: Rapid imaging with refocused echoes
SPE: Solid phase extraction SSTR: Somatostatin receptor
TE: Echo time
TR: Repetition time
TRNT: Targeted radionuclide therapy
U(H)PLC: Ultra (high) pressure liquid chromatography
