# Supplementary material for: Automated GMP compliant production of [18F]AlF-NOTA-octreotide
Source: EJNMMI Radiopharm Chem. 2020 Jan 29;5:4. doi: 10.1186/s41181-019-0084-1 (PMC6989705; doi:10.1186/s41181-019-0084-1)
Supplement: Supplementary file 1 — Additional file 1: Figure S1. Representative chromatogram (220 nm) of the formulation solution (blank). Figure S2. Radiochromatogram of [18F]AlF-NOTA-octreotide. A) Analytical chromatogram of [18F]AlF-NOTA-octreotide at the end of synthesis. Figure S3. In vivo biodistribution of [18F]AlF-NOTA-octreotide in control and blocking (coinjection with 2.5 mg/kg octreotide) [file 41181_2019_84_MOESM1_ESM.docx]

Supplementary information

**Automated GMP compliant production of [^18^F]AlF-NOTA-octreotide**

Térence Tshibangu^1^, Christopher Cawthorne^2,3,4^, Kim Serdons^2^, Elin Pauwels^2,3^, Willy Gsell^4^, Guy Bormans^1^, Christophe M. Deroose^2,3^ and Frederik Cleeren^1^

*^1^ Radiopharmaceutical research, Department of Pharmaceutical and Pharmacological sciences, KU Leuven, Leuven, Belgium*

*^2^ Nuclear Medicine, University Hospitals Leuven, Leuven, Belgium*

*^3^ Nuclear Medicine and Molecular Imaging, Department of Imaging and Pathology, KU Leuven, Leuven, Belgium*

*^4^ Biomedical MRI/MoSAIC, Department of Imaging and Pathology, Biomedical Sciences Group, KU Leuven, Leuven, Belgium*

*Correspondence should be addressed to Guy Bormans (guy.bormans@kuleuven.be,* <https://pharm.kuleuven.be/radpharm>*)*


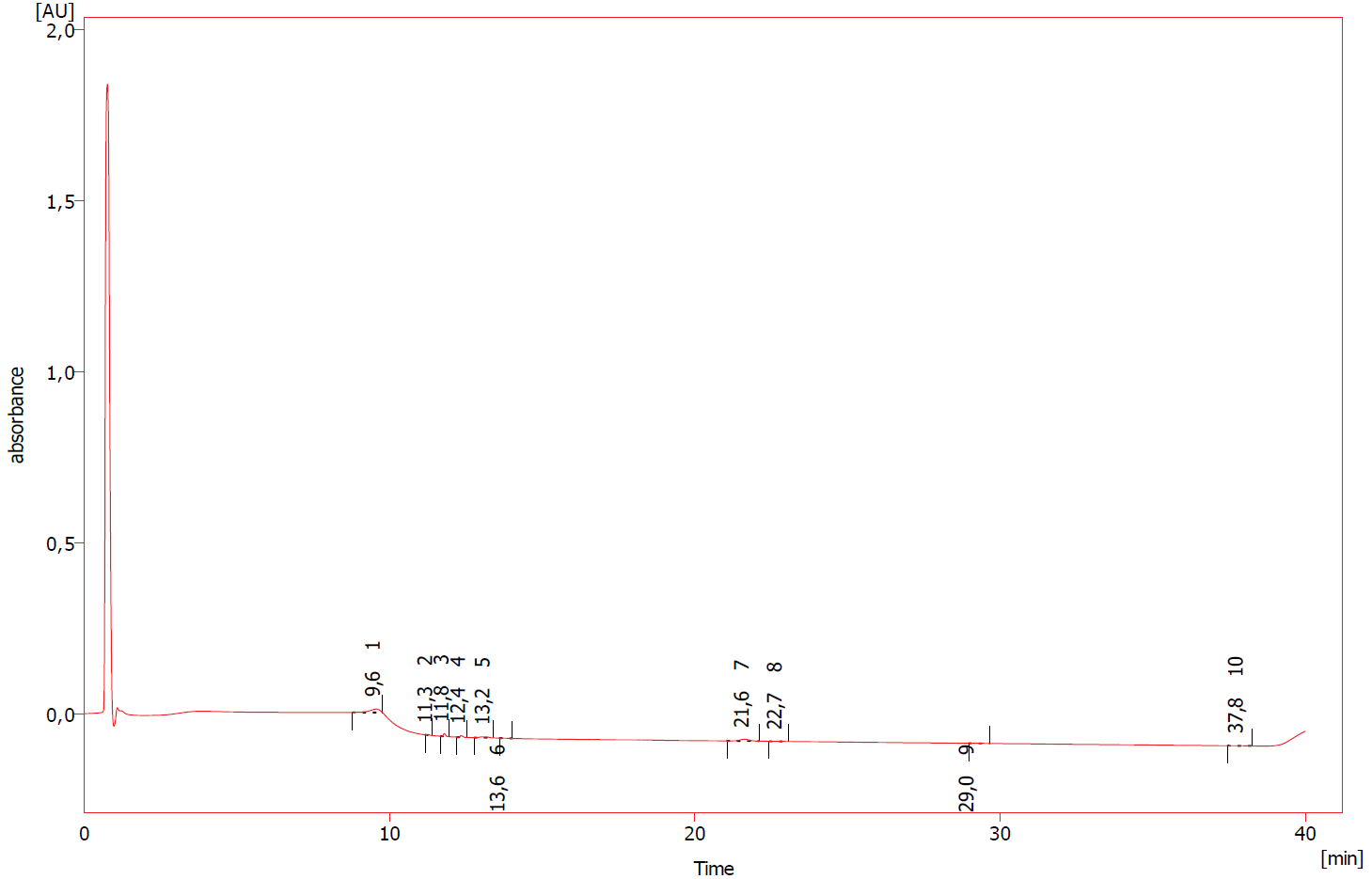


**Figure S1: Representative chromatogram (220 nm) of the formulation solution (blank)**

**
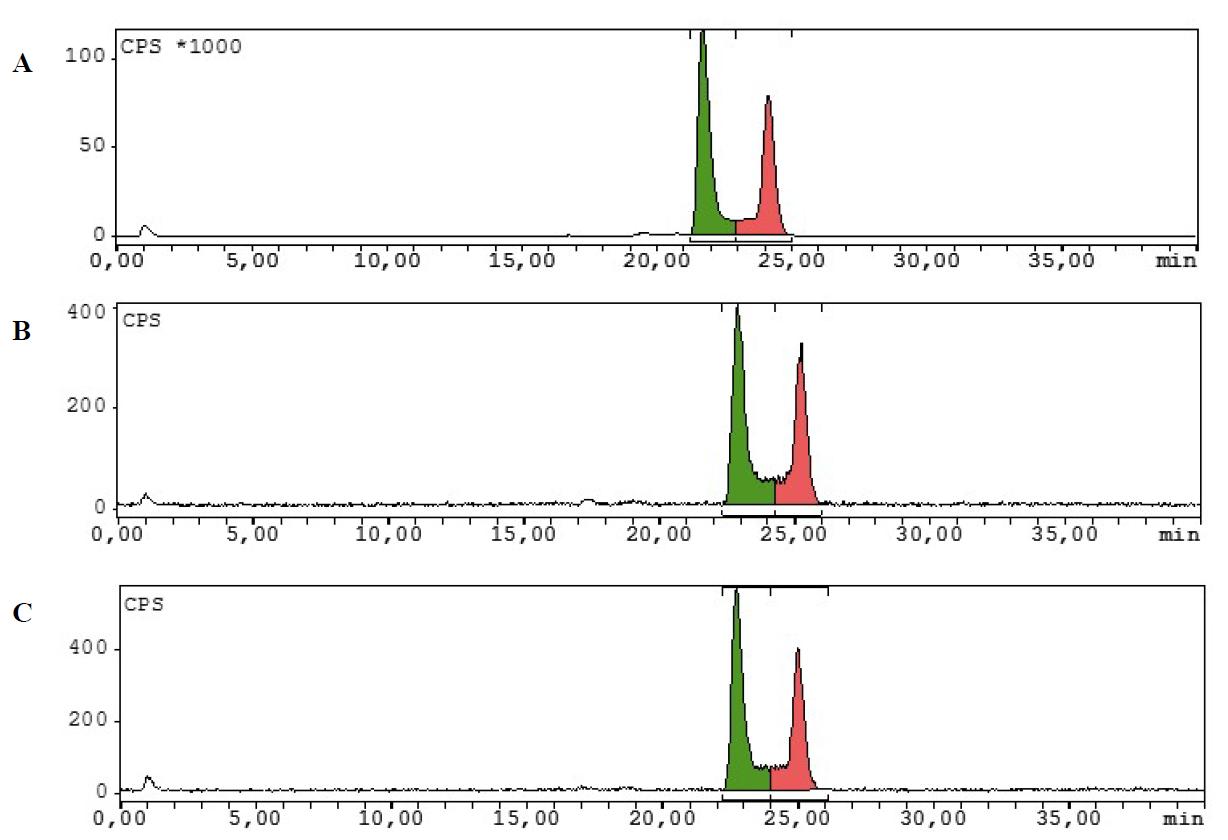
**

**Figure S2: Radiochromatogram of [^18^F]AlF-NOTA-octreotide. A) Analytical chromatogram of [^18^F]AlF-NOTA-octreotide at the end of synthesis,**  *Green peak and red peak correspond to two stereoisomer of [^18^F]AlF-NOTA-octreotide. Green peak and red peak were collected separately and reinjected on the HPLC system* **B)** **Analytical chromatogram of the first peak C)** **Analytical chromatogram of the second peak***. Reinjection of the collected peaks resulted again in two peaks in the radiochromatogram, suggesting that [^18^F]AlF-NOTA-octreotide isomers indeed undergo interconversion.*


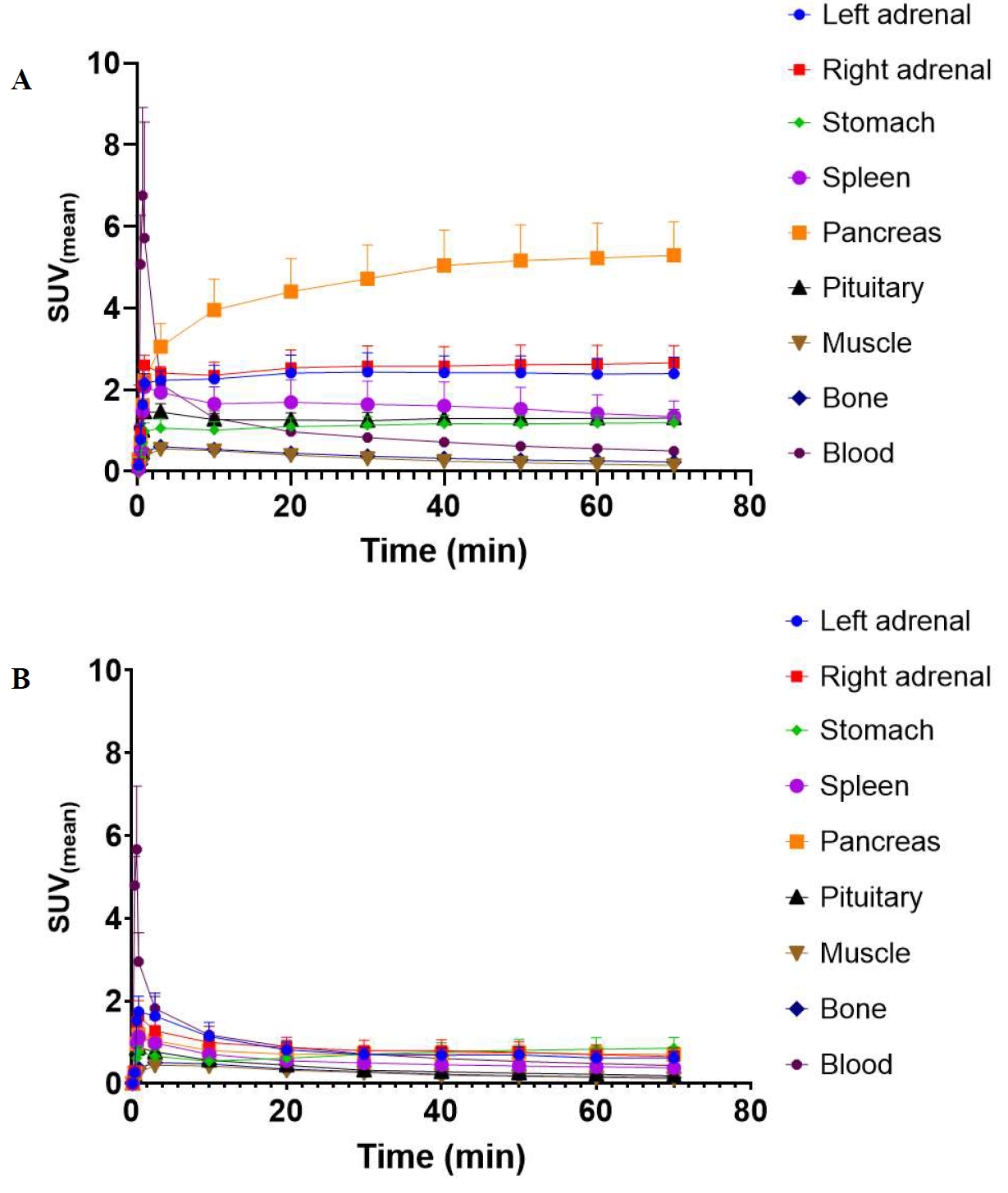


**Figure S3: *In vivo* biodistribution of [^18^F]AlF-NOTA-octreotide in control and blocking (coinjection with 2.5 mg/kg octreotide).** *Time activity curves of [^18^F]AlF-NOTA-octreotide uptake in naïve (A) and pre-blocked (B) animals. Data are reported as SUV_(mean)_ ± SEM*
